# Is Weight Gain Inevitable for Patients Trying to Quit Smoking as Part of Cardiac Rehabilitation?

**DOI:** 10.3390/ijerph17228565

**Published:** 2020-11-18

**Authors:** Ahmad Salman, Patrick Doherty

**Affiliations:** Department of Health Sciences, University of York, York YO10 5DD, UK; patrick.doherty@york.ac.uk

**Keywords:** cardiac rehabilitation, cardiovascular diseases, smoking, weight gain

## Abstract

The literature is uncertain about the extent to which those who attend cardiac rehabilitation (CR) gain weight while trying to quit smoking. This study aimed to determine the extent of CR-based smoking cessation provision and whether CR, as delivered in routine practice, is associated with helping patients quit smoking and avoid weight gain. Data from the UK National Audit of Cardiac Rehabilitation database, between April 2013 and March 2016, were used. Smoking status is categorised as smokers and quitters assessed by patient self-report. Outcomes included body weight, blood pressure, depression, and physical activity. A multiple linear regression model was constructed to understand the effect of continuing smoking or quitting smoking on CR outcomes. CR outcome scores were adjusted by the baseline CR score for each characteristic. An e-survey collected information about the smoking cessation support offered to patients attending CR. A total of 2052 smokers (58.59 ± 10.49 years, 73.6% male) and 1238 quitters (57.63 ± 10.36 years, 75.8% male) were analysed. Overall, 92.6% of CR programmes in the United Kingdom (UK) offer smoking cessation support for CR attenders. Quitting smoking during CR was associated with a mean increase in body weight of 0.4 kg, which is much less than seen in systematic reviews. Quitters who attended CR also had better improvements in physical activity status and psychosocial health measures than smokers. As delivered in routine practice, CR programmes in the UK adhere to the guideline recommendations for smoking cessation interventions, help patients quit smoking, and avoid weight gain on completion of CR.

## 1. Introduction

Smoking is a risk factor for cardiovascular disease (CVD) and the cause of death for approximately 8 million people annually [1]. For developing non-communicable diseases such as cardiovascular, cancers, and respiratory diseases, smoking is considered a preventable risk factor [2].

A meta-analysis by Aubin et al. of 62 clinical trials that described weight gain in smokers who quit smoking for up to 12 months suggests that body weight increased on average by 1.12 kg, 2.26 kg, 2.85 kg, 4.23 kg, and 4.67 kg at one, two, three, six, and 12 months, respectively, after quitting [3]. Most of the weight gain occurs within three months of quitting, and estimates of weight gain were similar among smokers using different pharmacotherapies to support smoking cessation [3].

A large systematic review and meta-analysis of 35 prospective cohort studies with 63,403 quitters and 388,432 continuing smokers looking at the association between smoking cessation and weight gain found that quitting smoking was associated with a mean weight gain of 4.10 kg and mean body mass index (BMI) gain of 1.14 kg/m^2^ over an average of 5 years [4]. The participants in this meta-analysis were similar to the general population in contrast to participants in the meta-analysis by Aubin et al. [3], making their findings more generalisable. In addition, the cohort studies in the meta-analysis by Tian et al. [4] had longer follow-up periods than those in the meta-analyses by Aubin et al. [3], which allows for an assessment of the effects of quitting smoking on weight change beyond 12 months.

The cross-sectional studies of the four EUROASPIRE surveys, which took place between 1999 and 2013, investigated characteristics of successful quitters who had been pre-event smokers and reported a non-smoking status at the time of interview (median 1.2 years [range 0.5 to 3 years]) [5]. They also found that smoking cessation was associated with weight gain [5]. Numerous cohort studies have shown that people who stop smoking gain weight [6,7,8,9,10,11,12].

Gaining weight while stopping smoking can lead to anxiety and depression. Systematic reviews and meta-analyses found that overweight, obesity, and depression interacted reciprocally and that overweight and obesity increased the risk for anxiety and depression [13,14,15].

Cardiac rehabilitation (CR) is a structured, multi-disciplinary intervention that is offered to patients with CVD with the goal of reducing risk factors (smoking) and promoting psychosocial wellbeing [16,17,18]. Positive health outcomes, such as a reduction in cardiovascular mortality and hospital readmission, have been associated with CR participation [19,20]. In the United Kingdom (UK), patients with CVD have access to CR programmes. Programme uptake in the UK averages 50% and is considered one of the highest uptake figures compared to other countries [21]. CR programmes are delivered to the British Association for Cardiovascular Prevention and Rehabilitation (BACPR) standards with the goal of reducing cardiovascular risk and promoting quality of life through coordinated core components of CVD prevention and rehabilitation [17]. Recommendations include providing smoking cessation support and relapse prevention through lifestyle risk factor management as one of its core components [17]. On average, 94% of individuals who join CR programmes in the UK are classified as non-smokers [21]. The average increase in smoking cessation among individuals who participate in CR is approximately one percent (1.1%) [21].

A key aim of CR and a goal for most patients is to bring the body mass index (BMI) below <30 kg/m^2^ [17]. On average, 30% of patients in the UK started CR with a BMI >30 kg/m^2^ [21]. The contribution of CR to reducing BMI at a national level is low, with an average change of 0.2% in patients with BMI <30 kg/m^2^ after CR [21]. This highlights the difficulty in addressing this risk factor. Additional factors need to be taken into account before drawing conclusions about how well CR programmes support weight management. Although smoking cessation results in considerable health improvements, it is often accompanied by weight gain, with patients who are trying to quit smoking more likely to put on 3–5 kg of weight in the first three months to a year [3]. Although weight gain does not offset the health benefits of smoking cessation, which far exceed any health risks that may result from smoking cessation-induced weight gain, it is frequently a source of concern for smokers planning to quit [7]. This substantial effect may inhibit the reporting of some successful weight loss programmes. The link between smoking and body weight is closely related and poses significant challenges for researchers investigating intervention effects in smokers. The most recent Cochrane review of 24 trials with a total of 7279 adult participants investigated the effectiveness of exercise-based interventions alone, or combined with a smoking cessation programmes and concluded no significant effect from adding exercise to smoking cessation [22]. The same authors do state and concludes that more studies are needed and that future trials may alter these conclusions [22]. Moreover, new research published in the British Journal of Pharmacology has confirmed that exercise can help smokers quit smoking and may aid smoking cessation by reducing the severity of smoking withdrawal symptoms [23]. As weight gain may be a barrier to quitting smoking or a reason to restart smoking, CR has not been evaluated in relation to weight gain after smoking cessation. As weight gain may be a barrier against quitting smoking, it is important to investigate smoking cessation support services provided in CR to help patients quit smoking while maintaining their weight status. Little is known about how routinely delivered CR programmes support smoking cessation.

This study determines the extent of CR-based smoking cessation provision and whether CR, as delivered in routine practice, is associated with helping patients quit smoking and avoid weight gain.

## 2. Materials and Methods

### 2.1. Data Source

Individual, patient-level data from the National Audit of Cardiac Rehabilitation (NACR) were used in the analyses of this study. The NACR, funded by the British Heart Foundation, is a web-based registry of CR in the UK. Data on patients who are eligible or referred to CR delivery are entered by practitioners into an electronic patient dataset according to a data dictionary (www.cardiacrehabilitation.org.uk/nacr/downloads.htm). The NACR team checks data quality from clinical teams who directly enters data into a secure online system (hosted by NHS Digital) who then provide NACR with anonymized local programme-level data. The NACR includes details of a patient’s initiating event, treatment type, risk factors, drugs, patient demographics, and post-CR clinical outcomes. Anonymised data is collected for a range of clinical indicators for the purposes of audit and research under Section 251 of the NHS Act 2006 [21]. NHS Digital annually reviews data governance and approval for NACR projects that aim to improve service quality and patient outcomes. Separate approval for this study in addition to the e-survey project was not required, as it was considered part of the NACR quality and outcomes process. The Strengthening the Reporting of Observational Studies in Epidemiology (STROBE) guidelines were used for reporting this observational study [24].

### 2.2. Participants

Participants included in this study were from the research cohort added to the NACR database between 1 April 2013 and 31 March 2016. Data has been validated and extracted retrospectively and there were no exclusion criteria. Analyses included patient sociodemographic data and clinical characteristics of individuals who started a CR programme with both a baseline and follow-up smoking status assessments.

### 2.3. Smoking Outcome Measures

Smoking status is assessed via self-reported questionnaires in the NACR database [21]. Pre- and post-CR smoking status is categorised into one of the following: never smoked, ex-smoker, stopped smoking since the event, or current smoking. For the purposes of this study, patients were categorised as continued smokers (defined as current smokers in pre- and post-CR assessments) or quitters (defined as current smokers in pre- and no smoking status in post-CR assessment).

A range of patient-level variables collected by the NACR primary dataset were used for the present study. Variables included anthropometric data, physical, and psychosocial health measures. Anthropometric measures included weight (kg), height (m), body mass index (BMI) (kg/m^2^), and waist circumference (cm). Alcohol consumption status was measured using self-reported questionnaires related to weekly alcohol consumption. Physical activity was self-reported and categorised into moderate physical activity (150 min/week; yes/no) or vigorous physical activity (75 min/week; yes/no)) using the Chief Medical Officer’s Physical Activity Questionnaire [25]. Physical activity recommendations were based on the Department of Health guidelines for 19–64 and 65+ age groups. The Hospital Anxiety and Depression Scale (HADS), a reliable and well-validated scale, was used to assess psychosocial health status. Higher HADS scores represent worse symptoms.

### 2.4. e-Survey

With the knowledge that smoking cessation is a key part of secondary prevention and rehabilitation and is included in the BACPR core components of lifestyle risk factor management [17], a cross-sectional 11-item e-survey was sent to CR services to explore smoking cessation services provided by CR programmes in the UK. The sampling frame encompassed the ‘coordinators’ of the 224 CR programmes in the UK that electronically enter their data into the NACR. Several reminders were sent out via email over two months. Data collection took place in the summer of 2016 (May 2016–July 2016). The response rate was 78% (175/224 CR programmes registered in the NACR).

### 2.5. Statistical Analysis

All analyses were performed in the IBM Statistical Package for Social Sciences (SPSS) software statistics Version 24 (New York, NY, USA). A *p*-value < 0.05 was considered statistically significant.

Percentage or relative change was used to measure the difference in outcome (post-CR) from baseline (pre-CR) [26,27]. It was calculated by: (percentage change = pre-CR value − post-CR value/pre-CR value) * 100

Outliers were detected by the median plus or minus 3 times the median absolute deviation (3 ± MAD) method [28]. Pre-and post-CR values with more than 3 ± MAD percentage change for each characteristic were eliminated from the analysis.

A multiple linear regression model was constructed to understand the effect of continuing smoking or quitting smoking on CR outcomes, with adjustments for the outcome CR score by the baseline CR score for each characteristic. Post-CR outcomes (with respect to baseline) were introduced into multiple linear regression models (as continuous dependent variables) and tested against smoking status (a score of 0 was categorised as a smoker, whereas a score of 1 was categorised as a quitter). We compared and described analyses of CR patients using the original data with analyses of all data after replacing missing values, which were handled through the expectation maximisation method [29]. Use of expectation maximisation to handle missing data gave similar results to the original analyses. Commonly used descriptive statistical parameters, including the number of programmes, percentages, means or medians, and standard deviations, were used to explore the data.

## 3. Results

### 3.1. Cohort Characteristics

A total of 49,725 patients had a pre- and post-CR smoking status recorded. Non-smokers comprised 93.4% of the sample (mean age 65.72 ± 11.08 years, 74.7% male), while 4.1% of the sample were classified as continued smokers (mean age 58.59 ± 10.49 years, 73.6% male) and 2.5% were quitters (mean age 57.63 ± 10.36 years, 75.8% male). The median duration of CR was 9 weeks. For the purposes of this research, patients were categorised as continued smokers or quitters (Table 1).

### 3.2. Smokers versus Quitters (Outcomes)

The CR outcome results between smokers and quitters are summarised in Table 2.

After controlling for baseline characteristics, predictions were made to determine outcome changes for patients who quit smoking while attending CR. Only CR patients with pre- and post-CR values were included in the analysis after excluding pre- and post-values with percentage change more than 3 ± MAD.

A multiple regression model was constructed to understand the effect of quitting smoking on CR outcomes with adjustments for the outcome CR score by the baseline CR score for each characteristic (Table 3). Moreover, post-CR outcomes (with respect to baseline) were introduced into multiple linear regression models (as continuous dependent variables) and tested against smoking status (score 0 for smokers; score 1 for quitters).

A χ^2^ test was conducted for the association between smokers and quitters and moderate physical activity (150 min/week) outcomes: improved (n = 679), no change (n = 1126), and worsened (n = 93). There was a statistically significant association between the smoking group and moderate physical activity outcomes: χ^2^(2) = 23.50, *p* < 0.001, and small association Cramér’s V = 0.11 (Table 4). A χ^2^ test was conducted for the association between smokers and quitters and vigorous physical activity (75 min/week) outcomes: improved (n = 338), no change (n = 1217), and worsened (n = 47). There was a statistically significant association between smoking status and vigorous physical activity outcomes: χ^2^(2) = 17.88, *p* < 0.001; small association Cramér’s V = 0.11) (Table 4).

### 3.3. e-Survey

**Figure 1:**
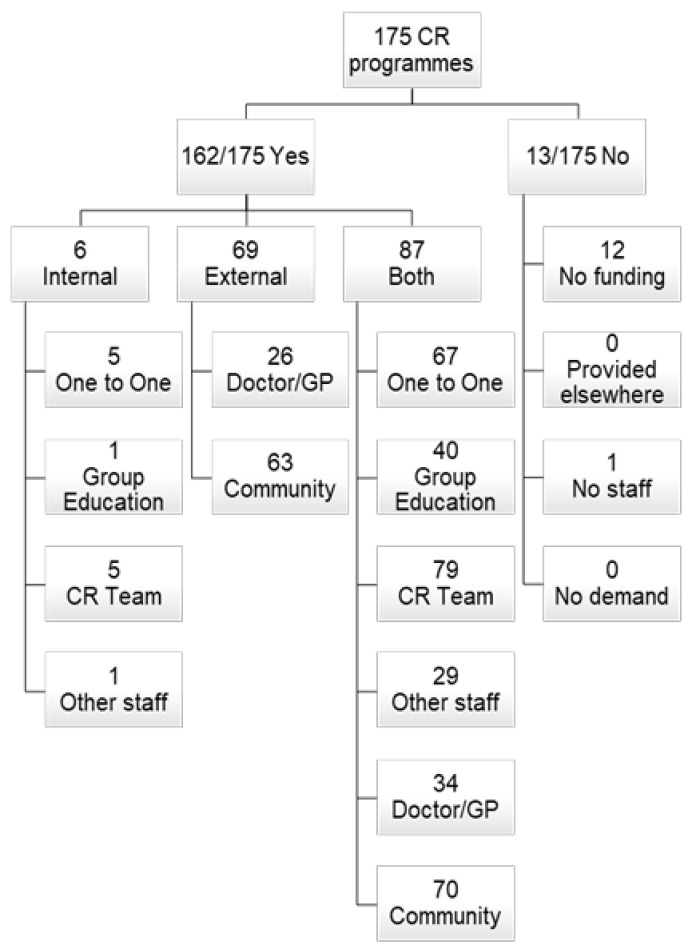
Number of cardiac rehabilitation programmes providing stopping smoking support. CR: cardiac rehabilitation; Internal: delivering the smoking cessation support services at the CR programme site; External: external referral.

Overall, 175 CR programmes participated—a response rate of 78% (175/224 CR programmes registered in the NACR). The following results present an overview of the survey results (Figure 1).

Most CR programmes in the UK offered smoking cessation support for CR attenders: 162 (92.6%) programmes, while 13 (7.4%) of CR programmes did not provide patients with support to stop smoking.

About half of CR programmes (87 (49.7%) programmes) offered both internal and external smoking cessation support for CR attenders. Six CR programmes only offered internal support, by delivering smoking cessation support services at the CR programme sites, while 69 (39.4%) CR programmes only offered external referrals.

Notably, 72/93 (77.4%) CR programmes that delivered smoking cessation support at the CR programme sites (internal delivery: 6 only internal + 87 both = 93 internal) offered one-to-one sessions. On the other hand, 41 (44.1%) CR programmes offered group education support as a form of internal support.

Eighty four (90.3%) CR programmes that offered smoking cessation support delivered it internally through the CR team. On the other hand, 30 (32.3%) CR programmes delivered smoking cessation support through other qualified staff members.

Sixty (38.5%) CR programmes that offered external support for smoking cessation (external delivery: 69 only external + 87 both = 156 external) offered referrals to doctors or general practitioners and 133/156 (85.3%) CR programmes offered referrals to community-based cessation programmes.

For 73/162 (45.1%) CR programmes that offered smoking cessation support, patient preference was the most frequently cited factor for whether a patient attended an internal CR programme’s smoking cessation service or was referred to external support (Table 5). However, eight (4.9%) CR programmes cited availability as a factor that influenced whether a patient would receive internal or external support; one (0.6%) reported funding constraints, and 36 (22.2%) CR programmes reported specific patient needs (e.g., hardened smoker).

Funding was the most common factor for not providing support for smoking cessation for CR attenders, given as the reason by 12/13 (92.3%) CR programmes that did not provide support for patients to stop smoking. The other factor reported by only one CR programme was lack of appropriate staff.

## 4. Discussion

Our research findings show that, after CR, quitters, on average, gain 0.43 kg more than those who continue to smoke (*p* < 0.001) and have a BMI of 0.18 kg/m^2^ more than those who continue to smoke (*p* < 0.001). Although differences in weight and BMI scores after CR were statistically significantly different for quitters and continued smokers (driven by a large sample size of 49,725), the mean differences of 0.43 kg and 0.18 kg/m^2^ were very small from a clinical perspective and much lower than previously cited reviews of smoking cessation where the mean weight gain was around 4 kg [3,4,5]. The lack of clinically relevant differences in this data are sufficient to make a strong clinical recommendation regarding the impact of CR to prevent weight gain when delivered alongside smoking cessation in patients with heart disease. Our study shows no clinically significant weight gain in the short term.

Evidence suggests that quitting smoking is associated with a mean increase in body weight of 3–5 kg, with most weight gain occurring within 3 months of quitting [3,4,5]; however, the research findings reported here show that smokers who quit smoking while attending CR do not gain weight, which aligns with the findings of Farley et al. that exercise could reduce post-cessation weight gain [30]. With regard to smoking and weight interactions, the extent of weight gain associated with smoking cessation in patients attending CR is much less than previous studies suggest. These research findings provide evidence that CR is positively associated with weight management during smoking cessation.

The confidence interval for mean difference in weight between continued smokers and quitters after CR was 0.22 to 0.63 kg and for mean difference in BMI, it was 0.1 to 0.25 kg/m^2^. Because of the well-documented health benefits of quitting smoking, clinicians should inform smokers about the low likelihood of weight gain and encourage them to attend CR to avoid excess weight gain.

There is no clinical trial evidence for the effectiveness of smoking cessation interventions within CR; however, our research findings suggest CR as delivered in routine practice is associated with helping patients quit smoking and reduce the likelihood of weight gain beyond 1 kg. The NACR data regarding smoking status suggest that about 37.6% of patients who are smoking when recruited to CR successfully stop after CR. Quitting smoking is considered a core element in both primary and secondary prevention of cardiovascular disease [31].

Following CR, quitters on average drink 1.34 units of alcohol fewer than those who continue to smoke. Following CR, 43% and 26.6% of quitters improved to achieve the recommended UK moderate and vigorous physical activity guidelines, respectively, compared with 31.9% and 18% of continued smokers. An even stronger benefit was seen in both HADS anxiety and depression scores, which showed that quitters on average score 0.75 and 0.58 less anxious and less depressed than those who continue to smoke.

Our survey of smoking cessation support services, offered in routine practice to CR attenders, had a high response rate of 78%. Although one study has shown low levels of cessation support following hospital discharge [32], the e-survey showed that 92.6% of CR programmes in the UK offer smoking cessation support for patients attending CR. These results show that CR programmes in the UK adhere to guideline recommendations for smoking cessation interventions [17,33,34,35]. In addition, the research results suggest that CR programmes in the UK offer assistance for patients who smoke by delivering smoking cessation support at the CR programme site in the form of individualised one-to-one sessions or group educational sessions, as well as referral for external smoking cessation support. The internal support is provided by the CR team or another qualified member of staff. One-to-one sessions are the dominant service offered at the site of CR programmes, while external provision is predominantly through referral to community-based cessation programmes. Patient preference is the factor that most influences whether a patient would attend the CR programme (internal) or be referred out (external).

Provision of smoking cessation support in CR could have multiple benefits: the presence of such a programme could entice more smokers to attend CR, and the increased support for cessation they receive could encourage them to remain in the CR programme generally. Prior studies suggest that CR attendance improves smoking cessation rates, and Riley et al. found a strong relationship between smoking cessation and CR attendance [36].

Failure of adherence to guideline recommendations to provide support for smoking cessation for CR attenders was predominantly due to funding challenges. Cutting funds to CR services is a false economy, as evidence shows that smoking cessation services provide effective support for smokers who want to quit [37] and the lack of this provision leads to higher costs for the NHS to manage and treat diseases caused by smoking in the long term. The National Institute for Health and Care Excellence (NICE) estimates that for every pound invested in smoking cessation, £2.37 in benefits are generated [38]. Moreover, the lack of investment in CR programmes may impact on service provision. In Yorkshire, for example, a qualitative study found staff to be aware of limited service availability [39], which may influence which patients are invited. Finally, it should not have to be a choice that some smokers attending CR are supported to quit and others are not.

Comprehensive CR programmes seem to have a beneficial role in helping patients after a cardiac event or procedure, with significant improvements in smoking behaviour, weight management, physical activity levels, psychosocial health, and alcohol consumption. When a comprehensive CR includes exercise with smoking cessation and patient education, this research initiates evidence for improvements in cardiac risk factors, particularly increased smoking cessation and improvements in physical and psychosocial health.

Several limitations of our study must be noted. First, the retrospective observational study design is limited in capturing data and data quality from CR programmes. An 18% gap in data capture was identified due to some programmes not providing their data electronically. Approximately 31% of patients who began a CR programme did not have post-program data available, affecting data quality. Non-completion of the program leads to missingness in patient records that may affect the representativeness of our sample. Self-reported data poses a limitation in terms of determining smoking status and immediate post-CR analysis. Using self-reported data to determine smoking status might be subject to recall and social desirability biases. The self-reported smoking status was not validated with a biochemical marker. Some possible factors that influenced quitting smoking have been missed from the analysis due to high levels of missing data or may not have been collected in the NACR. Some smoking cessation drugs in addition to the intensity of the smoking cessation program (number and duration of the visits) may have affected the considered outcomes and they are not included as a confounder in the analyses for the outcomes related to smoking cessation and weight gain [40,41,42]. However, the strengths of our study include utilizing a prospective cohort design and an observational approach, and using data from a large-scale dataset that collects routine clinical information from CR programmes in the UK. This study also suggested that clinical and research efforts should be directed towards improving the rate of smoking cessation in patients with CVD by accounting for factors that predict quitting smoking among CR attendees [43].

## 5. Conclusions

Cardiac rehabilitation is an effective intervention to manage weight gain when quitting smoking. Quitting smoking during CR is associated with a mean increase of 0.4 kg in body weight following CR. Quitters who attended CR improved their physical activity status and psychosocial health measures compared with smokers.

This research is the first to evaluate smoking cessation support in CR services in the UK, with 92.6% of CR programmes in the UK offering smoking cessation support for CR attenders. These results demonstrate adherence of CR in the UK to the guideline recommendations for smoking cessation interventions.

## Figures and Tables

**Table 1:** Smoking categorisation groups.

Group	Frequency (n)	Percent (%)
Smokers	2052	62.4
Quitters	1238	37.6
Total	3290	100

n = Number of patients; % percentage of patients.

**Table 2:** Baseline and outcome values for cardiac rehabilitation (CR) patients included in the analysis.

CR Outcome	Smokers			Quitters		
	Pre-CR	Post-CR	n	Pre-CR	Post-CR	n
Weight (Kg)	81.64	81.68	1499	83.83	84.28	881
BMI (kg/m2)	27.99	28.28	1442	28.01	28.47	833
Waist (cm)	98.47	98.09	657	97.39	97.11	272
Alcohol consumption	17.78	13.80	486	15.66	11.28	298
HADS anxiety score	7.89	7.39	1046	6.92	5.79	546
HADS depression score	6.53	5.68	1032	5.44	4.24	530

BMI, body mass index; CR, cardiac rehabilitation; HADS, hospital anxiety and depression scale; n = number of patients.

**Table 3:** Summary of multiple regression analysis.

Variable (N)		Unstandardised Coefficients		Standardised Coefficients		95% CI		Effect Size
		B	S.E.	Beta	Sig.	Lower	Upper	
Weight (n = 2380)	Constant	0.75	0.24		<0.001	0.28	1.23	0.01
	Baseline weight	0.99	0.00	0.99	<0.001	0.99	1.00	
	Smoking	0.43	0.11	0.01	<0.001 *	0.22	0.63	
BMI (n = 2275)	Constant	0.41	0.10		<0.001	0.22	0.61	0.01
	Baseline BMI	0.99	0.00	0.99	<0.001	0.98	0.99	
	Smoking	0.18	0.04	0.02	<0.001 *	0.10	0.25	
Waist (n = 929)	Constant	4.52	0.75		<0.001	3.05	5.99	0.00
	Baseline waist	0.95	0.01	0.97	<0.001	0.94	0.97	
	Smoking	0.05	0.23	0.00	0.83	-0.40	0.49	
Alcohol consumption (784)	Constant	3.86	0.54		<0.001	2.80	4.91	0.01
	Baseline alcohol consumption	0.56	0.02	0.73	<0.001	0.52	0.60	
	Smoking	−1.34	0.68	-0.05	0.05 *	−2.68	0.00	
HADS anxiety score (1592)	Constant	0.86	0.16		<0.001	0.56	1.17	0.02
	Baseline HADS anxiety score	0.77	0.02	0.76	<0.001	0.74	0.80	
	smoking	−0.75	0.15	-0.08	<0.001 *	−1.04	-0.45	
HADS depression score (1562)	Constant	0.64	0.14		<0.001	0.37	0.91	0.01
	Baseline HADS depression score	0.74	0.02	0.74	<0.001	0.70	0.77	
	smoking	-0.58	0.14	-0.07	<0.001*	-0.86	-0.30	

B = unstandardised regression coefficient; Beta = standardized coefficient; BMI, body mass index; CI = Confidence Interval for unstandardised regression coefficient; CR, cardiac rehabilitation; HADS, hospital anxiety and depression scale; n = Number of patients; S.E. = standard error of the coefficient. * *p* < 0.05.

**Table 4:** Summary of multiple regression analysis.

Physical Activity Outcomes	Smokers (%)			Quitters (%)		
	Improve	No Change	Worsen	Improve	No Change	Worsen
∆ 150 min/week (moderate)	31.9	62.8	5.4	43	52.9	4.1
∆ 75 min/week (vigorous)	18.0	79.3	2.6	26.6	70.0	3.5

∆, change; % percentage.

**Table 5:** What might decide whether a patient would attend the CR Programme or be referred out?

Reason	n = 162	Percentage (%)
Availability	8	4.9
Patient preference	73	45.1
Service funding constraints	1	0.6
Specific patient needs	36	22.2

n = Number of programmes; % percentage of programmes.
